# Preoperative Prediction of the Scaphoid Screw Length for the Fixation of Fractures Using MRI Measurements of Finger Phalanges

**DOI:** 10.7759/cureus.73805

**Published:** 2024-11-16

**Authors:** Rudra M Prabhu, Prashant Kamble, Sameer Panchal, Himanshu Choudhury, Shubhranshu S Mohanty, Muhammad Mannan, Nayan Shrivastava, Muhammad A Hamid

**Affiliations:** 1 Orthopedics, University Hospitals Birmingham NHS Foundation Trust, Birmingham, GBR; 2 Orthopedics, Seth Gordhandas Sunderdas Medical College and King Edward Memorial Hospital, Mumbai, IND; 3 Orthopedics, Wirral University Teaching Hospital NHS Foundation Trust, Upton, GBR; 4 Radiology, Sir H. N. Reliance Foundation Hospital and Research Centre, Mumbai, IND

**Keywords:** middle phalanx, mri, preoperative planning, ring finger, scaphoid fracture, screw length

## Abstract

Introduction: Scaphoid fractures are one of the most common carpal bone fractures, with most fractures involving the waist. When surgery is indicated, internal fixation with screws is the standard method for the fixation of these fractures. Accurate length and trajectory of the screw are two crucial parameters essential for optimal internal fixation. Determining the accurate screw length required for fixation can be challenging, especially in an intraoperative setting. Preoperative determination of the screw length can improve surgical precision, thereby contributing to an improved functional outcome. This study investigates a novel approach using magnetic resonance imaging (MRI) measurements of the little, ring, middle, and index finger middle phalanges to preoperatively predict the screw length used for scaphoid fracture fixation.

Methods: This retrospective observational study included an analysis of 30 MRIs of the wrist and hand. The MRI protocol for wrist and hand was unified for all the patients to ensure consistency and reproducibility. All MRIs were performed by a single experienced radiologist using a high-field 1.5 Tesla MRI scanner. The axial lengths of the bones were calculated on T1-weighted coronal MRI slices using the Meddiff Rispacs DICOM viewer software. A line was drawn from the condyles of the phalanx to the base of the phalanx to measure the maximum axial length of the middle phalanx. The maximum axial length of the scaphoid was measured from the proximal pole to the distal articular surface along the fracture fixation axis. The measurements were performed by a consultant orthopedic hand surgeon and a qualified orthopedic senior resident to minimize inter-observer errors. A musculoskeletal radiologist further analyzed the measurements. Pearson’s correlation coefficient was used to determine the relationship between the scaphoid length and the lengths of the middle phalanges. Paired t-tests were applied with the level of significance set at p < 0.05. Linear regression was performed to develop a predictive equation for scaphoid length based on the middle phalanx measurements.

Results: The mean scaphoid length was 1.96 cm. The mean lengths of the middle phalanges of the little, ring, middle, and index fingers were 1.66 cm, 2.05 cm, 2.29 cm, and 1.79 cm, respectively. Using the paired t-test, a significant positive correlation was found between the scaphoid length and middle phalanx length of all fingers (p < 0.001), with the strongest correlation observed with the length of the middle phalanx of the ring finger (r = 0.861). The regression equation for predicting scaphoid length based on the middle phalanx of the middle finger was: y = 0.665 + 0.565x (study power 90%).

Conclusion: MRI measurements of the middle phalanx, particularly of the ring finger, offer a reliable method for predicting scaphoid screw length preoperatively. This approach can improve surgical planning, precision, and potentially patient outcomes without increasing additional costs.

## Introduction

Scaphoid fractures account for 60% of carpal fractures, most occurring at the waist, followed by the distal and proximal thirds [1]. Internal fixation with a screw to compress and stabilize the fracture is the preferred modality of management when surgical intervention is indicated. Surgical management has been shown to decrease morbidity and allow a faster return to work. Accurate screw length and screw trajectory are vital factors required for a favorable functional outcome [2]. The screw length required for fixation could be determined by measuring the length of the other scaphoid bone on a plain radiograph. However, because of the scaphoid's twisted profile, assessment of length using plain radiographs is difficult [3]. Particularly when percutaneous fixation is chosen, preoperative knowledge of the required screw length would aid in planning the surgery, reducing operating time, and increasing procedural efficiency. The use of MRI for confirming scaphoid fractures has increased in recent times due to the limitations of plain radiographs, with scaphoid fractures often being missed on plain radiographs. A wrist MRI that has been done to confirm the scaphoid fracture could also include imaging of the hand, allowing a comparison of the length of the middle phalanges of the fingers and the scaphoid. If a relationship between the length of the middle phalanges and the scaphoid is proven, the scaphoid length can be known by measuring the length of the middle phalanges on the same side. Our study aimed to determine this relationship with the help of MRI.

## Materials and methods

**Figure 1:**
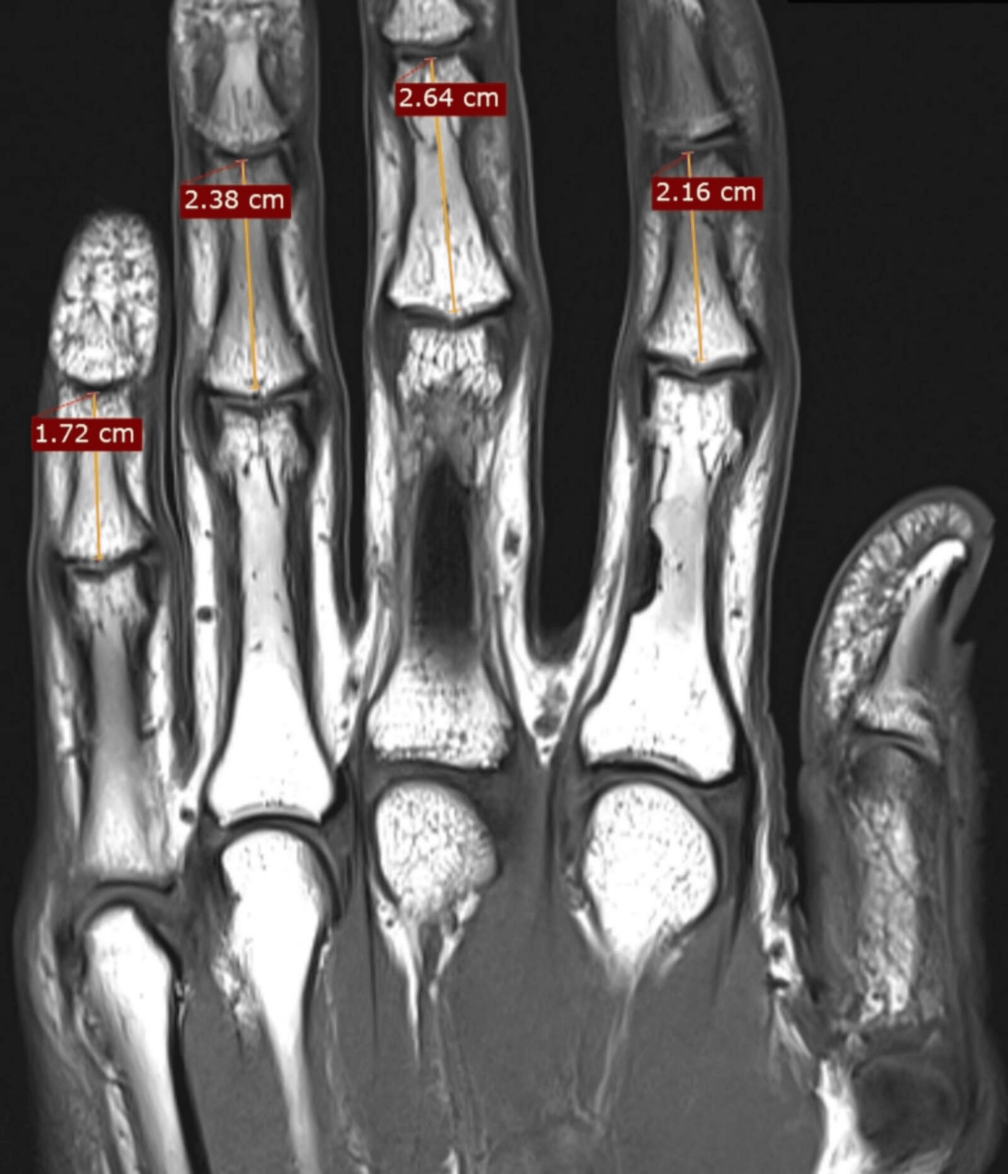
Technique showing the measurement of the length of the middle phalanx on coronal T1-weighted MRI sequences.

The present study received approval from the Institutional Review Board of Seth Gordhandas Sunderdas Medical College and King Edward Memorial Hospital. This retrospective observational study analyzed 30 MRIs performed between November 2020 and November 2021. The MRIs included in the study were normal MRIs that were performed to diagnose suspected wrist or hand pathologies. Patients with any evidence of injury, arthritis, congenital anomaly, and previous surgery of the wrist and hand were excluded. A unified MRI protocol was used for all patients to ensure consistency and reproducibility. MRIs were performed using a high-field 1.5 Tesla MRI scanner (Siemens Healthineers, Germany). The maximum axial length of the middle phalanx was measured from the condyles of the phalanx to its base (Figure 1).

**Figure 2:**
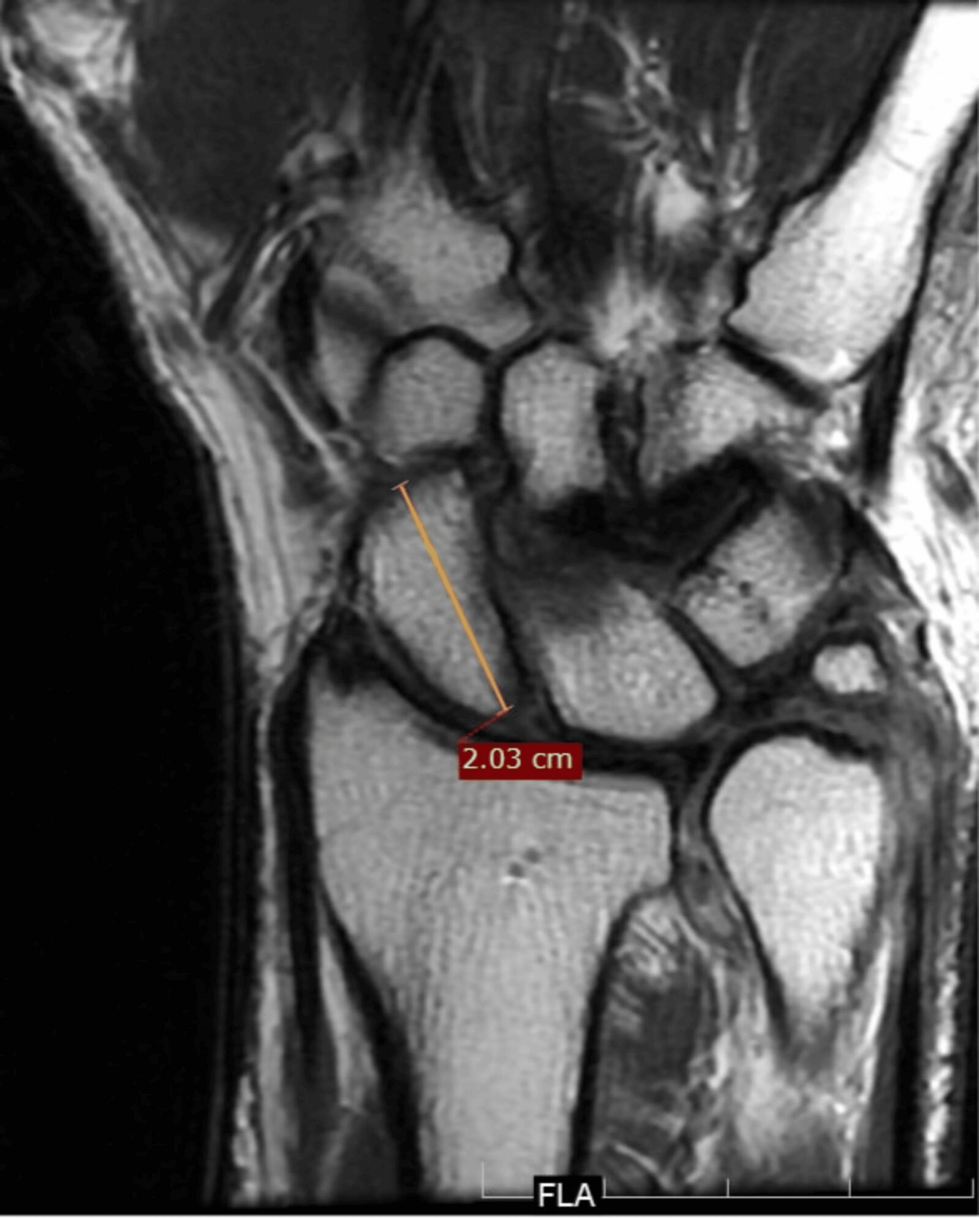
Technique showing the measurement of the scaphoid length on coronal T1-weighted MRI sequences.

The maximum axial length of the scaphoid was measured from the proximal pole to the distal articular surface along the fracture fixation axis (Figure 2).

**Figure 3:**
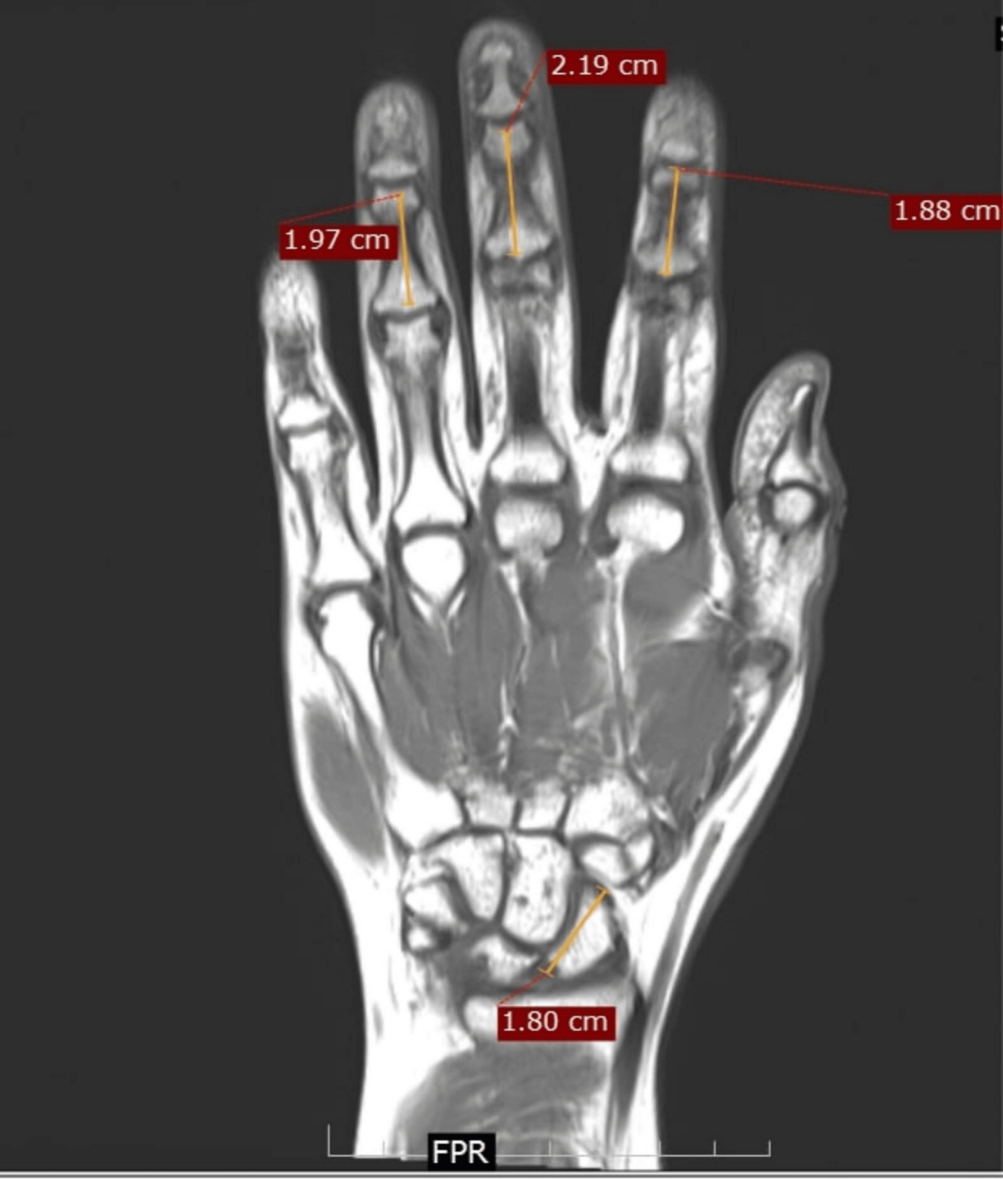
Coronal T1-weighted MRI section of the hand and the wrist showing the method to measure the length of the middle phalanges and the scaphoid.

The lengths of the phalanges and the scaphoid could be compared in the sequences that included the carpals and the phalanges (Figure 3).

To minimize the intraobserver error, the measurements were performed on three different images of the same MRI, and the average of those measurements was taken into consideration. The measurements were performed on coronal T1-weighted images using the Meddiff Rispacs DICOM viewer software (Meddiff Technologies Pvt Ltd, India) by a consultant orthopedic hand surgeon and a qualified orthopedic senior resident to minimize the inter-observer errors. The measurements were further analyzed by a dedicated musculoskeletal radiologist. Pearson’s correlation coefficient was used to determine the relationship between the scaphoid length and the lengths of the middle phalanges. Paired t-tests were applied with the level of significance set at p < 0.05. Linear regression was performed to develop a predictive equation for scaphoid length based on the middle phalanx measurements.

## Results

Analysis using the paired t-test showed that there was a significant relationship between the maximum axial length of the scaphoid and the middle phalanges of the little, ring, middle, and index fingers (p < 0.001). Further analysis involved determining the correlation coefficients for each finger. This showed that the relationship was strongest between the scaphoid and the middle phalanx of the ring finger (Table 1).

**Table 1:** Comparison of the lengths of the middle phalanges of the little, ring, middle, and index fingers and the scaphoid. SD: standard deviation; MP: middle phalanx *:significant p-value (<0.05)

	Mean (SD)	Pearson’s correlation coefficient	p-value
Scaphoid length	1.96 (0.228)		-
MP of little finger	1.66 (0.199)	0.693	<0.001*
MP of ring finger	2.05 (0.251)	0.861	<0.001*
MP of middle finger	2.29 (0.291)	0.722	<0.001*
MP of index finger	1.79 (0.210)	0.663	<0.001*

On the left side, significant correlations were found between the length of the scaphoid and the length of the middle phalanges of the ring, middle, and index fingers. On the right side, significant correlations were observed between the length of the scaphoid and the length of the middle phalanges of all four fingers, with the correlation again being the strongest for the ring finger on both sides (Table 2).

**Table 2:** Correlation between the scaphoid length and the length of the middle phalanges for each side. MP: middle phalanx *: significant p-value (<0.05)

Left side			Right side	
Pearson’s correlation coefficient	p-value		Pearson’s correlation coefficient	p-value
0.744	0.055	MP of little finger	0.662	0.001*
0.879	0.009*	MP of ring finger	0.851	<0.001*
0.777	0.040*	MP of middle finger	0.692	<0.001*
0.814	0.026*	MP of index finger	0.614	0.002*

A linear regression model was developed to predict the scaphoid length based on the middle phalanx length of the middle finger. The resulting equation was:

Axial length of the scaphoid (y) = 0.665 + 0.565 × length of the middle phalanx of the middle finger

The power of the study was calculated to be 90%, indicating a high probability that the study detected a true effect where one existed.

## Discussion

In patients with radial-sided wrist pain post-trauma, a scaphoid fracture is the most common diagnosis [4]. However, these fractures can often be missed on initial radiographs. It can sometimes take around six weeks for a scaphoid fracture to be detected on a plain radiograph, with around 40% of fractures missed at the initial presentation [5]. The American College of Radiology suggests initial radiography for patients with suspected scaphoid fractures. If radiographs are negative, the next step is to immobilize the wrist, followed by repeat radiographs after 10 to 14 days. Wherever possible, an early MRI can be performed in cases where a fracture is clinically suspected but not detected on radiographs. This would enable earlier surgical intervention if a fracture is detected and needs to be managed surgically [6]. Early MRI use in suspected scaphoid fractures has been associated with lesser pain and higher satisfaction rates [7]. Imaging the bones of the hand along with the wrist could aid in measuring the axial length of the middle phalanges of the fingers on MRI sequences. This would help to predict the screw length required for fracture fixation. The present study hypothesized that a strong correlation exists between the axial length of the scaphoid and the axial length of the middle phalanges of the little, ring, middle, and index fingers. This correlation would aid the surgeon in the preoperative planning of scaphoid fractures.

The use of early MRI to detect occult scaphoid fractures has been shown to reduce potentially high costs of unnecessary immobilization and allow early treatment [8,9]. Although cast immobilization with serial monitoring using radiographs is the preferred treatment in cases of occult or stable scaphoid fracture as per the Herbert classification [10], there is an increased risk of pseudo-arthrosis in cases where the immobilization is inadequate [11,12]. Moreover, immobilizing these fractures is a difficult task due to the complex biomechanics of the bone, leading to the requirement of an above-elbow cast and resultant elbow stiffness in some cases [13]. Surgical fixation offers the advantage of faster bone healing and a lesser duration of immobilization as compared to conservative treatment [14]. There have been a few studies in the literature that have investigated the morphology of scaphoids and correlated it with the screw length required for fixation. Heinzelmann et al. measured the morphology of scaphoids in cadaveric specimens and concluded that the usual screw length is 27 mm and 23 mm for male and female scaphoids, respectively, after allowing 2 mm countersinking of the screw beneath either pole [15]. The authors further stated that due to the smaller width of the proximal pole in the case of females, standard-sized screws may not be appropriate when planning insertion from the proximal to the distal direction. In this case, screws can be inserted from the distal to the proximal direction. Jain et al. performed measurements on cadaveric specimens and concluded that a correlation existed between the scaphoid’s axial length as measured by a vernier caliper and that of the ring finger’s middle phalanx on a radiograph [3]. They stated that the measurement of the required screw could be predicted by subtracting 4 mm from the middle phalanx axial length, and therefore, a radiograph imaging the wrist with the hand would aid in preoperative planning by enabling indirect assessment of the scaphoid’s axial length.

Jain et al. determined that there was a positive correlation between the axial length of the scaphoid and the axial length of the middle phalanges of all the fingers. The correlation was better with the ring finger on both sides, as determined by the correlation coefficient (r = 0.646). In the present study, the correlation coefficient, when comparing the scaphoid length and the length of the middle phalanx of the ring finger, was 0.861. When measurements comparing the length of the scaphoid and the middle phalanx were made for each side, the correlation coefficient was 0.879 and 0.851 for the left and the right side, respectively.

The primary objective of this study was to establish a reliable, indirect method for predicting the screw length for scaphoid fracture fixation using MRI measurements of the middle phalanges of the fingers. The findings of the current study suggest that preoperative MRI can enhance surgical planning by providing accurate estimations of the screw length needed for scaphoid fracture fixation. This is especially relevant for cases where intraoperative determination of screw length can be challenging, such as comminuted fractures of the scaphoid. By incorporating hand measurements during wrist MRI, surgeons can better prepare the necessary surgical tools, potentially reducing operative time and exposure to fluoroscopy and improving surgical outcomes. Current methods for determining screw length often rely on intraoperative measurements or plain radiographs, which can be inaccurate due to the scaphoid's complex geometry. Previous studies have highlighted the limitations of these approaches and the benefits of MRI in fracture detection and surgical planning. The present study adds to this body of knowledge by demonstrating a practical application of MRI for preoperative screw length prediction. The study ensured a unified MRI protocol was applied across all patients, conducted by a single experienced radiologist, which mitigates variability and enhances measurement accuracy. The study preferred the use of an MRI over a computed tomography (CT) scan due to the radiation exposure associated with the latter. Although a CT scan would help to exclude the cartilage, unlike an MRI, the associated radiation exposure did not warrant its use. While screw placement is crucial during the surgical fixation of scaphoid fractures, our study focuses on length prediction as a complementary tool. MRI not only helps in predicting screw length but can also detect associated ligamentous or soft tissue injuries, providing a more comprehensive assessment of the wrist. These injuries could be managed in the same surgical sitting used for fracture fixation.

The strengths of the current study included the use of a consistent and uniform MRI protocol and measurement process for all the participants. The primary limitation of the study is the sample size and its representation of the Indian population, which may affect the generalizability of the findings. The technique described here would provide us with the screw length for fixing fractures of the scaphoid waist, as the measurements were along the axis of that screw. As scaphoid waist fractures are the commonest, this technique could be utilized in the majority of cases. Larger studies with more diverse populations would help to confirm the reliability and accuracy of the predictive equation. Further research should aim to validate the predictive model. The existence of additional correlations could be determined by investigating whether similar correlations can be established with other bones or phalanges, enhancing the utility of the technique. Clinical outcomes of surgeries performed using this predictive method should be analyzed to determine its impact on patient recovery and surgical success rates.

## Conclusions

The study demonstrates that MRI can serve as a valuable adjunct in the preoperative planning of scaphoid fractures by enabling accurate prediction of screw length. This technique has the potential to improve surgical precision, reduce operative times, and enhance patient outcomes. By integrating this method into standard MRI protocols, clinicians can leverage existing imaging to gain critical preoperative insights without incurring additional costs.
